# Cytotoxic Flavonoid Glycosides from *Rapistrum rugosum* L.

**Published:** 2012

**Authors:** Areej Mohamed Al-Taweel, Ghada Ahmed Fawzy, Shagufta Perveen

**Affiliations:** a *Department of Pharmacognosy, College of Pharmacy, King Saud University, Riyadh, Kingdom of Saudi Arabia.*; b *Department of Pharmacognosy, Faculty of Pharmacy, Cairo University, Cairo 11562, Egypt. *

**Keywords:** SRB cytotoxic assay, *Rapistrum rugosum*, Flavonoid glycosides, *n*-butanol

## Abstract

Five flavonoid glycosides were isolated from the *n*-butanol soluble fraction of the ethanolic extract of *Rapistrum rugosum *and their structures were assigned from ^1^H- and ^13^C-NMR spectra (DEPT) with 2D NMR as quercetin-3-*O*-*α*-L-rhamnopyranoside (1), quercetin-3-*O*- *β*-D-xyloside (2), quercetin, 3-*O*-*α-*L-arabinopyranoside,7-*O*-*α*-L-rhamnopyranoside (3), kaempferol 3-*O*-*α-*L-arabinopyranoside, 7-*O*-*α*-L-rhamnopyranoside (4) and rutin (5). The SRB cytotoxic assay was used to investigate the antitumor activities of *n*-butanol extract, compound 3 and its hexaacetate 3a, for the first time. Compounds 3 and 3a showed cytotoxic activity against the human cancer cell line, namely, HepG2 (hepatocellular carcinoma cell line) with IC_50_ (concentration of compound required to reduce cell survival by 50%) 0.86 μg/mL and 3.50 μg/mL, respectively. These results proved that compound 3, the major flavonoid of the *n*-butanol soluble fraction, has significant cytotoxic activity compared with the standard antitumor drug doxorubicin (0.60 μg/mL).

## Introduction

*Rapistrum rugosum *L. commonly known as turnip weed, wild turnip or bastard cabbage, belongs to the family of Cruciferae. This family comprises about 390 genera and is represented in Saudi Arabia by 49 genera. *R. rugosum *is native to North Africa, Europe, the Middle East and Pakistan and is the only *Rapistrum *species in Saudi Arabia (1). The leaves of *R. rugosum *are externally applied to the heal legs furuncles in Italy (2). *R. rugosum *is boiled and used for culinary purposes as one of the most popular, wild food plants in Sicily (3). 

The high demand of the innovative lead structures to develop the novel drugs for the treatment of cancer and other menacing diseases drove us to study the cytotoxic activity of the ethanolic plant extract. The ethanolic extract of *R. rugosum *showed cytotoxic activity, on further biological screening of all fractions, the *n*-BuOH soluble fraction revealed a strong cytotoxic activity. This prompted us to carry out the phytochemical study and try to isolate the constituents of the *n*-BuOH soluble fraction of *R. rugosum*. Our goal was also to try and investigate the effect of acetylation on the cytotoxic activity of the major isolated compound. 

## Experimental

*General experimental procedures*

The ^1^H-, ^13^C-NMR, HMQC, and HMBC spectra were recorded on Bruker spectrometer operating at 400 and 100 MHz for ^1^H-NMR and ^13^C-NMR, respectively. The chemical shift values are reported in ppm (*δ*) unit and the coupling constants (*J*) are in Hz. The column chromatography was carried out on various adsorbents including silica gel 230-400 mesh, cellulose and sephadex LH-20 (E. Merck, Darmstadt, Germany). FAB and HRFABMS (neg. mode, matrix: glycerol) on JEOL JMS-HX110, mass spectrometer and Thin layer chromatography (TLC) were performed on precoated silica gel F_254_ plates (E. Merck, Darmstadt, Germany), silica gel RP-18 F_254_ and cellulose. The detection was done at 254 nm by spraying with ceric sulphate and AlCl_3_ reagents.

*Plant material*

The aerial parts of *R. rugosum *(1.00 Kg) were collected from Riyadh (Saudi Arabia) in February 2007 and identified by Dr. M. Atique Al-Rahman, Prof. of Taxonomy, College of Pharmacy, King Saud University, where a voucher specimen (No. 8) has been deposited.

*Extraction and isolation*

The air-dried powdered aerial parts of the plant (1.00 Kg) were subjected to the extract with 80% EtOH. The total extract (150 g) was suspended in water (250 mL) and successively partitioned with *n*-hexane (4 × 300 mL), chloroform (4 × 300 mL) and *n*-butanol (5 × 300 mL). The dried *n*-butanol extract (30 g) was subjected to fractionation on a Sephadex LH-20, using MeOH : H_2_O as eluent. Fractions were collected and grouped into (A-C) fractions through TLC analyses on silica 60 F_254_ gel-coated glass sheets developed with *n*-BuOH : AcOH : H_2_O (60 : 15 : 25).

Fraction A (3 g) was chromatographed on a Sephadex LH-20 column using *n*-BuOH/*iso-*pr.OH/H_2_O (BIW, 4:1:5, organic layer) for elution to afford 4 sub-fractions (I-IV). Sub-fraction II contained only one spot and it was then purified on a Sephadex LH-20 column with MeOH (eluent) to give compound 3 (75 mg). Sub-fraction III was chromatographed on a cellulose column with 80% MeOH/H_2_O as eluent and the collected fraction was further purified on Sephadex column with EtOH to yield compound 4 (15 mg).

Fraction B (1 g) was chromatographed on a Sephadex LH-20 column using *n*-BuOH/*iso-*pr.OH/H_2_O (BIW, 4 : 1 : 5, organic layer) for elution to afford 3 sub-fractions (I-III). Sub-fraction I contained only one spot and it was then purified on a Sephadex LH-20 column with MeOH: H_2_O (2 : 8) as eluent to give compound 5 (13 mg).

Fraction C (1.5 g) was a binary mixture which was separated on a cellulose column with 80% MeOH/H_2_O to afford 1 (15 mg) and 2 (12 mg).

*Acetylation of compound 3 (3a)*

Compound 3 (20 mg) was dissolved in pyridine-acetic anhydride (1:1, 2 mL) and stirred overnight at room temperature. The mixture was evaporated to dryness at rotary evaporator in vacuum under N_2_ which gave a hexaacetate of 3 as white amorphous powder. HRFABMS (-ve ion mode): *m/z *831.0625 calcd. for C_38_H_39_O_21_, 831.0633.

*Acid Hydrolysis of compound 3*

Compound 3 (5 mg) in MeOH (5 mL) containing 1N HCl (5 mL) was refluxed for 4 h, concentrated under reduced pressure and diluted with H_2_O (10 mL). It was extracted with EtOAc and the residue recovered from the organic phase yielded quercetin as an aglycone. The remaining aqueous solutions were evaporated to dryness, resolved in MeOH and subjected to TLC analysis (eluent: EtOAc-MeOH-H_2_O-HOAc, 6:2:1:1). The chromatogram was sprayed with aniline hydrogen phthalate followed by heating at 100°C. The sugars were identified after the comparison through authentic standards.

*Cytotoxicity assay*

*In-vitro SRB cytotoxic assay against Human liver cancer cell line (HepG2 cells)*

Potential cytotoxicity of *n*-butanol extract, compounds 3 and 3a were tested using the method of Skehan and Storeng (4). The sensitivity of the human tumor cell lines to thymoquinone was determined through the SRB assay. SRB is a bright pink aminoxanthrene dye with two sulphonic groups. It is a protein stain that binds to the amino groups of intracellular proteins under mildly acidic conditions to provide a sensitive index of cellular protein content. Hepatocellular cell line (HepG2) was obtained frozen in liquid nitrogen at -180°C from the American Type Culture Collection and the tumor cell line was maintained in the National Cancer Institute, Cairo, Egypt, through serial sub-culturing. The cell lines were grown in monolayer cultures in RPMI 1640 medium (Sigma Chemical Co., St. Louis, Mo, U.S.A) supplemented with 10% Fetal Bovine Serum (FBS) (Sigma Chemical Co., St. Louis, Mo, U.S.A), Penicillin/Streptomycin (Sigma Chemical Co., St. Louis, Mo, U.S.A): 100 units/mL Penicillin and 2 mg/mL Streptomycin and maintained at 37°C in a 5% CO_2_/95% air atmosphere, with 95% humidity. Cells were plated in 96-multiwell plate (10^4^cell/well) for 24 h before the treatment with tested compounds to allow the attachment of cell to the wall of the plate. Different concentrations of the tested compounds (1, 2.5, 5 and 10 μg/mL) were added to the cell monolayer. Monolayer cells were incubated with compounds for 48 h at 37°C in 5% CO_2_ atmosphere. After 48 h, cells were fixed, washed and stained with sulforhodamine B (SRB) stain (Sigma Chemical Co., St. Louis, Mo, U.S.A. 0.4 % SRB dissolved in 1 % acetic acid was used as a protein dye). Excess stain was washed with acetic acid and the attached stain was recovered with Tris-EDTA buffer. Color intensity was measured in an ELISA reader (Meter tech. Σ 960, U.S.A.) at λ _max_ 564 nm. The mean background absorbance was automatically subtracted and the mean values of each drug concentration were calculated. The relation between the surviving fraction and compound concentration was plotted to get the survival curve of the tumor cell line after the specified tested compounds. Furthermore, the IC_50_ of each tested compound was calculated using Graph-Pad PRISM program (Graph-Pad, UK) (5). The standard antitumor drug used was doxorubicin.

## Results and Discussion

The mixture of flavonoid glycosides obtained from the *n*-BuOH fraction of the ethanolic extract of *R. rugosum *was subjected to a series of column chromatographic separations to isolate compounds 1-5, namely; quercetin-3-*O*-*α*-L- rhamnopyranoside (1), quercetin-3-*O*-*β*-D-xyloside (2), quercetin, 3-*O*-*α-*L-arabinopyranoside,7-*O*-*α*-L-rhamnopyranoside (3), kaempferol 3-*O*-*α-*L-arabinopyranoside, 7-*O*-*α*-L-rhamnopyranoside (4) and rutin (5). Their structures were established via mass and NMR spectroscopy including 2D NMR techniques and through comparison with the reported data in the literature (6-13).

*Quercetin; 3-O-α-L-arabinopyranoside, 7-O-α-L-rhamnopyranoside (3*)

*Yellow crystalline solid (75 mg), M.p. 248-250ºC*: EIMS *m/z *(rel. int.): 302 (100), 270 (10), 152 (27), 134 (28). HRFABMS (-ve ion mode): *m/z *579.0625 calcd. for C_26_H_27_O_15_, 579.0633. ^1^H-NMR (400 MHz, DMSO-*d*_6_) δ: 6.44 (1H, d, *J *= 2.0 Hz, H-6), 6.78 (1H, d, *J *= 2.0 Hz, H-8), 6.82 (1H, d, *J *= 8.0 Hz, H-5′), 7.56 (1H, d, *J *= 2.2 Hz, H-2′), 7.70 (1H, dd, *J *= 8.0, 2.2 Hz, H-6′), 5.56 (1H, brs, H-1′′′), 5.30 (1H, d, *J *= 4.5 Hz, H-1′′), 3.76 (1H, dd, *J *= 8.4, 4.7 Hz, H-2′′), 3.53 (1H, m, H-3′′), 3.63 (1H, m, H-4′′), 3.24 (1H, m, H-5a′′), 3.62 (1H, m, H-5b′′), 3.5 (1H, m, H-2′′′), 3.3 (1H, m, H-3′′′), 3.1 (1H, m, H-4′′′), 3.2 (1H, m, H-5′′′), 1.12 (3H, d, *J *= 5.5 Hz, H-6′′), ^13^C-NMR (100 MHz, DMSO-*d*_6_) δ: 156.7 (C-2), 133.8 (C-3), 177.6 (C-4), 160.8 (C- 5), 98.4 (C-6), 161.6 (C-7), 94.3 (C-8), 156.8 (C-9), 105.5 (C-10), 120.7 (C-1′), 115.8 (C-2′), 145.0 (C-3′), 148.8 (C-4′), 115.2 (C-5′), 122.1 (C-6′), 101.2 (C-1′′), 70.2 (C-2′′), 71.5 (C-3′′), 65.9 (C-4′′), 64.2 (C-5′′), 99.4 (C-1′′′), 70.3 (C-2′′′), 70.4 (C-3′′′), 71.6 (C-4′′′), 70.0 (C-5′′′), 17.8 (C-6′′′).

**Figure 1:**
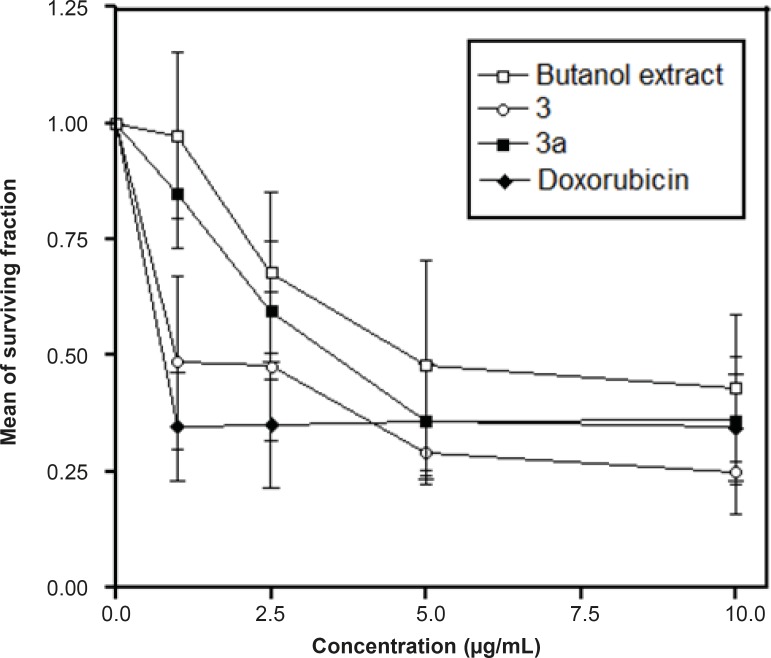
The effects of different concentrations of the *n*-butanol extract, compounds 3 and 3a on HepG2 cell survival as assessed through the SRB Cytotoxic Assay

**Figure 2:**
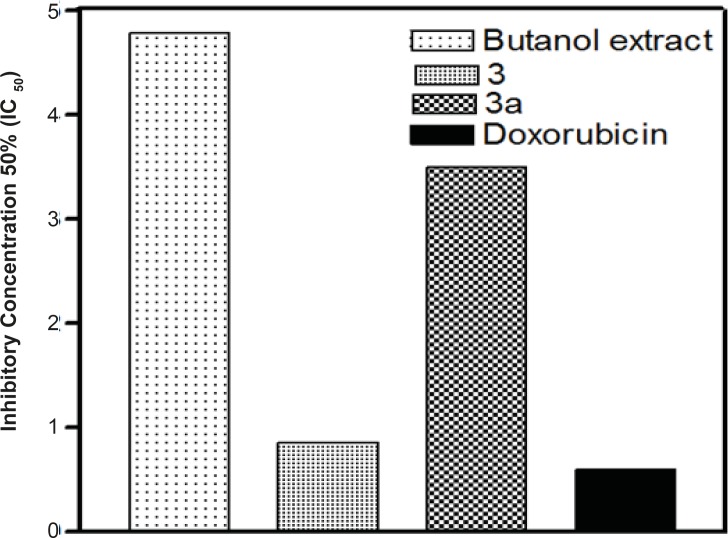
The IC_50_-values of n-butanol extract, compounds 3 and 3a

The major compound 3 was derivatized into its hexaacetate derivative (3a) and analyzed through HRFABMS and NMR. The major flavonoid diglycoside (3) and its hexaacetate (3a) were screened for cytotoxicity against the human cancer cell line, namely, HepG2 (hepatocellular carcinoma cell line). From the results shown in Table 1 and Figures 1 and 2, it could be seen that compound 3 shows a significant cytotoxic activity against the liver carcinoma cell line (IC_50_ = 0.86 μg/mL), while the acetylated compound 3a shows a lower cytotoxic activity (IC_50_ = 3.50 μg/mL) compared to the standard drug doxorubicin (IC_50_ = 0.60 μg/mL).

**Table 1:** Effect of *n*-butanol extract, compounds 3 and 3a on liver carcinoma cell line (HepG2). Mean of surviving fraction ± SD, n = 6

**Tumor cell line**	**Extract and compounds conc. μg/mL**	***n*** **-butanol extract**	**Compound 3**	**Compound 3a**	**Doxorubicin ** ^c^
HepG2	0.000	1.000 ± 0.000	1.000 ± 0.000	1.000 ± 0.000	1.000 ± 0.000
	1.000	0.973 ± 0.180^a^	0.485 ± 0.186	0.847 ± 0.115 ^a^	0.347 ± 0.117
	2.500	0.678 ± 0.172 ^a^	0.477 ± 0.161	0.597 ± 0.149	0.350 ± 0.136
	5.000	0.479 ± 0.227	0.291 ± 0.070	0.358 ± 0.117	0.359 ± 0.124
	10.000	0.430 ± 0.159	0.249 ± 0.089	0.360 ± 0.136	0.345 ± 0.115
^b^IC_50_		4.78 μg/mL	0.86 μg/mL	3.50 μg/mL	0.60 μg/mL

^a^Significantly different from standard drug at p ≤ 0.01, using ANOVA and Tukey-Kramer multiple comparisons tests.^b^IC_50_: concentration of compound required to reduce the cell survival by 50%.^c^Doxorubicin: standard antitumor drug

**Figure 3:**
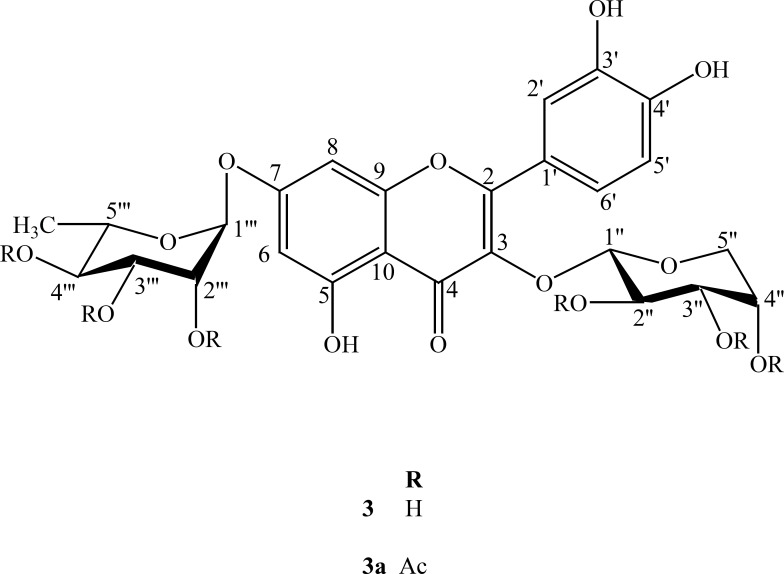
Structure of compounds 3 and 3a

Quercetin-3-*O*-*α*-L-rhamnopyranoside (1), quercetin-3-*O*-*β*-D-xyloside (2), quercetin, 3-*O*-*α-*L-arabinopyranoside,7-*O*-*α*-L- rhamnopyranoside (3), kaempferol 3-*O*-*α-*L-arabinopyranoside, 7-*O*-*α*-L-rhamnopyranoside (4) and rutin (5) have been isolated for the first time from *Rapistrum*. By means of chemical methods and spectroscopic analyses, the structures of these compounds (1-5) were established. There were no previous published reports dealing with the NMR data of the flavonoid diglycoside 3. 

Compound 3 was obtained as a yellow crystalline solid. The HRFABMS of 3 exhibited a pseudomolecular ion peak [M-H]^-^ at *m/z *579.0625 (calcd. for C_26_H_27_O_15_, 579.0633) consistent with the molecular formula of C_26_H_27_O_15_. The EIMS spectrum showed different peaks of aglycone at *m/z *302, 270, 154 and 150. The UV spectrum exhibited characteristic absorption maxima for a flavonoid glycoside at λ_max_ (nm), (MeOH): 260, 300 (sh). The EIMS gave a peak at *m/z *302 due to successive losses of sugar moieties. The ^1^H NMR spectrum displayed a signal at *δ *12.50 for a chelated hydroxyl group and two meta coupled protons of ring A at *δ *6.44 (d, *J *= 2.0 Hz) and 6.78 (d, *J *= 2.0 Hz). It further showed three aromatic protons of ring B forming an ABX system at *δ *6.82 (d, *J *= 8.0 Hz, H-5), *δ *7.56 (d, *J *= 2.2 Hz, H-2) and *δ *7.70 (dd, *J *= 8.0, 2.2 Hz, H-6′). In the aliphatic region, an anomeric proton signal at δ 5.30 (d, *J *= 4.5 Hz), together with two oxymethylene protons observed at δ 3.24 (m) and δ 3.62 (m) were indicative to the presence of *α*-L-arabinopyranoside moiety. The second anomeric proton was observed at δ 5.56 as (brs). The ^1^H NMR spectrum further showed signals of oxymethine protons in the range of δ 3.76-3.10 and the methyl protons resonated at *δ *1.12 (d, *J *= 5.5 Hz) which was characteristic for *α*-L-rhamnopyranoside moiety. 

The ^13^C NMR and DEPT spectra showed twenty-six signals comprising one methyl, one methylene, fourteen methine and ten quaternary carbons. The signals at *δ *156.7, 133.8, 177.6 and 105.5 were typical of C-2, C-3, C-4 and C-10 of a flavonol moiety. The signals of two anomeric carbons of the sugar moieties appeared at *δ *101.2 and 99.4. Assignment of all ^1^H and ^13^C resonances was proved through their comparison with the reported data in the literature (6-13). Acid hydrolysis of 3 provided L-arabinose and L-rhamnose and it was confirmed through the TLC of sugars with their standards.

In addition, the major flavonoid diglycoside (3) and its acetylated form (3a) were screened for cytotoxicity against the human cancer cell line, namely, HepG2 (hepatocellular carcinoma cell line).

Previous studies, however, proved the antitumor activity of flavonoids and even aimed at elucidating the structure-activity relationships in order to develop new anticancer drugs (14). This is the first report for the cytotoxic activity of these compounds. This finding may help to show the structural requirements implicated in the anticancer activity of flavonoids, with the goal of rationalizing their development as antitumor agents.
